# Pentoxifylline Sensitizes Cisplatin-Resistant Human Cervical Cancer Cells to Cisplatin Treatment: Involvement of Mitochondrial and NF-Kappa B Pathways

**DOI:** 10.3389/fonc.2020.592706

**Published:** 2020-12-16

**Authors:** Alejandro Bravo-Cuellar, Pablo Cesar Ortiz-Lazareno, Erick Sierra-Díaz, Fabiola Solorzano-Ibarra, Anibal Samael Méndez-Clemente, Adriana Aguilar-Lemarroy, Luis Felipe Jave-Suárez, Édgar Ruiz Velazco-Niño, Georgina Hernández-Flores

**Affiliations:** ^1^ División de Inmunología, Centro de Investigación Biomédica de Occidente (CIBO), Instituto Mexicano del Seguro Social (IMSS), Guadalajara, Mexico; ^2^ Departamento de Ciencias de la Salud, Centro Universitario de los Altos, Universidad de Guadalajara, Tepatitlán de Morelos, Mexico; ^3^ Departamento de Urología, Hospital de Especialidades, Centro Médico Nacional de Occidente, Instituto Mexicano del Seguro Social (IMSS), Guadalajara, Mexico; ^4^ Programa de Doctorado en Ciencias Biomédicas Orientación Inmunología, Centro Universitario de Ciencias de la Salud (CUCS), Universidad de Guadalajara (UdeG), Guadalajara, Mexico

**Keywords:** pentoxifylline, cervical cancer cells, chemoresistance, cisplatin, NF-KappaB

## Abstract

**Background:**

Cervical cancer continues to be a major public health problem worldwide, and Cisplatin is used as first-line chemotherapy for this cancer; however, malignant cells exposed to CISplatin (CIS) become insensitive to the effects of this drug. PenToXifylline (PTX) is a xanthine that sensitizes several types of tumor cells to apoptosis induced by antitumor drugs, such as Adriamycin, Carboplatin, and CIS. The effects of PTX on tumor cells have been related to the disruption of the NF-κB pathway, thus preventing the activation of cell survival mechanisms such as the expression of anti-apoptotic genes, the secretion of proinflammatory interleukins, and growth factors.

**Objective:**

In this work, we studied the antitumor proprieties of PTX in human SiHa cervical carcinoma cells resistant to CIS.

**Materials and Methods:**

SiHa and HeLa cervical cancer cells and their CIS-resistant derived cell lines (SiHaCIS-R and HeLaCIS-R, respectively) were used as *in-vitro* models. We studied the effects of PTX alone or in combination with CIS on cell viability, apoptosis, caspase-3, caspase-8, and caspase-9 activity, cleaved PARP-1, anti-apoptotic protein (Bcl-2 and Bcl-xL) levels, p65 phosphorylation, cadmium chloride (CdCl_2_) sensitivity, Platinum (Pt) accumulation, and glutathione (GSH) levels, as well as on the gene expression of GSH and drug transporters (influx and efflux).

**Results:**

PTX sensitized SiHaCIS-R cells to the effects of CIS by inducing apoptosis, caspase activation, and PARP-1 cleavage. PTX treatment also decreased p65 phosphorylation, increased Pt levels, depleted GSH, and downregulated the expression of the *ATP7A*, *ATP7B*, *GSR*, and *MGST1* genes.

**Conclusion:**

PTX reverses the acquired phenotype of CIS resistance close to the sensitivity of parental SiHa cells.

## Introduction

Cervical cancer is a health problem worldwide and is the most frequent cause of cancer-related death in women despite advances in screening, prevention, and treatment (1). The prognosis of patients with advanced/recurrent cervical cancer is particularly poor, and their chance of a 1-year relative survival rate is only 10–20% (2, 3). Cisplatin *cis-diamminedichloroplatinum* (*II*) is the principal chemotherapeutic agent used in the treatment of a wide variety of solid tumors (4). CISplatin (CIS) is the most effective drug against cervical cancer in neoadjuvant and salvage therapy, but its administration is severely hindered by the occurrence of resistance. The development of intrinsic and acquired drug resistance in cancer after cycles of treatment is relatively common and remains a major challenge for CIS-based anticancer therapy. There are many mechanisms underlying CISplatin-Resistance (CIS-R). However, increasing drug efflux, elevated intracellular glutathione levels (detoxification), inhibition of apoptosis, and alterations in the expression of BCL-2 family members are the most consistent characteristics that contribute to the CIS-R phenotype in cervical cancer (5, 6). Resistance to chemotherapy has been reported to promote tumor cell progression and metastasis (7). Thus, it is important to develop novel therapeutic modalities. Based on the mechanisms underlying CIS-R, the administration of CIS, in combination with other drugs, has been proposed to overcome resistance in cervical cancer. The pharmacological reduction of resistance is a preferred approach in targeting malignant cells and can provide new directions in cancer therapy based on the concept of chemotherapy with a rational molecular basis (8). We have previously reported that PenToXifylline (PTX) in combination with CIS significantly increases the induction of apoptosis and inhibits cell proliferation by suppressing Nuclear Factor kappa B (NF-κB) in human cervical cancer cells (9). PTX is a xanthine and a non-specific Phosphodiesterase inhibitor, which can act as a potent tumor necrosis factor alpha (TNF-α) inhibitor and can reduce inflammation through the inhibition of IκB phosphorylation in serines 32 and 36 (8, 10, 11). It has been demonstrated that PTX, in combination with some antitumor drugs, significantly increases cell apoptosis in several types of human cancer cell lines, such as cervical cancer cells (HeLa and SiHa) (9), retinoblastoma cells (Y79) (12), leukemia cells (U937) (13), and breast cancer cells (MCF-7 and MDA-MB-231) (14). Additionally, our earlier studies demonstrated that L5178Y lymphoma-bearing mice treated with PTX + ADRyamicin (ADR) survived more than 1 year after receiving only one half of the standard therapeutically active ADR dose, compared to single treatments of ADR (8). In the clinic, PTX has demonstrated to induce remission and increase apoptosis in pediatric patients with acute lymphoblastic leukemia during the steroid-window phase (15). Moreover, PTX enhanced the action of Gemcitabine in the treatment of pancreatic xenograft tumors in a BALB/c-nu/nu mouse model (16). Although PTX is reported to be an efficient sensitizing agent, its cytotoxic effect against CIS-resistant cervical cancer cells has not, to our knowledge, been described previously. Therefore, the aim of the present study was to determine the effect of PTX, either alone or in combination with CIS, on human cervical carcinoma cells with acquired resistance to CIS.

## Methods

### Cell Culture Lines

The human cervical cancer cell lines used in this study were SiHa (Human Papilloma Virus, HPV16^+^) and HeLa (HPV18+). They were kindly provided by Prof. Frank Roesl (DKFZ, Heidelberg, Germany). Both cell lines were authenticated utilizing the Multiplex Cell Authentication system by Multiplexion GmbH (Friedrichshafen, Germany). The presence of the HPV type was confirmed by the Linear Array HPV Genotyping test (Roche). These cell lines were cultured in Dulbecco’s Modified Eagle’s Medium (DMEM; GIBCO Invitrogen, Carlsbad, CA, USA), supplemented with 10% heat-inactivated fetal bovine serum and antibiotics (Penicillin-Streptomycin; GIBCO Invitrogen). The medium is referred to as DMEM-S. The cells were grown at 37°C in a humidified atmosphere of 95% air and 5% CO_2._ The cells were passaged once they reached 75–85% confluence. Prior to the initiation of all experiments, cell viability was determined with Trypan Blue (Sigma-Aldrich, St. Louis, MO, USA) (viability >95%). The cell lines were tested for mycoplasma contamination employing the Universal Mycoplasma Detection Kit (ATCC, Manassas, VA, USA); the cells were negative throughout the study.

### Drugs

CIS was purchased from PISA Laboratories, Guadalajara, Jalisco, Mexico. A stock solution (400 µM) of CIS was prepared by dissolving the powder in a 0.9% NaCl solution and was stored at −4°C in the dark. PTX (Sigma-Aldrich, St. Louis, MO, USA) was dissolved in the DMEM-S culture medium to obtain a 250-mM stock solution and was stored at −4°C. Cadmium chloride (CdCl_2_; Sigma-Aldrich, St. Louis, MO, USA) was dissolved in type-1 Milli-Q^®^ water and then filtered using 0.45-µm filters. A 500-µM stock solution was then prepared in DMEM-S. The different solutions were stored separately prior to performing the experiments in cervical cancer cells and were discarded after that they were utilized in each assay.

### Establishment of Cisplatin-Resistant Human Cervical Cancer Cell Lines

To induce CIS resistance in parental HeLa and SiHa cell lines (HeLaP and SiHaP), the cells were exposed repeatedly to increasing concentrations of CIS during 12 months (17). First, the cells were exposed to 0.01 µM CIS; subsequently, the concentration of CIS was increased every four passages, from 1, 10, 20, 50, 75, to 200 µM CIS. The resulting CIS-resistant cells were denominated HeLaCIS-R and SiHaCIS-R, respectively. To maintain the CIS phenotype, these cells were grown in DMEM-S culture medium with CIS at 1 μM.

### Experimental Conditions

Human cervical cancer cell lines were seeded into flasks and maintained in DMEM-S culture medium to 80–90% confluence. The cells were then harvested with the Accutase^®^ Cell Detachment solution (GIBCO Invitrogen Corp.) and were seeded (10 × 10^6^) in 150-mm tissue culture dishes to evaluate Platinum (Pt) content, GSH, Poly (ADP-Ribose) Polymerase (PARP-1), Bcl-2, Bcl-XL, the phosphorylation of NF-κB/p65, and caspase-3, -8, and -9 activity. To assess cytotoxicity, viability, and apoptosis (DNA fragmentation), 2 × 10^4^ cells/well/200 µl (final volume) were cultured in 96-well plates. For the evaluation of different genes (*ATP7A*, *ATP7B*, *CTR1*, *MRP-2*, *GSR*, *GSS*, *MGST1*, *GPX*, and *RPL32*) by quantitative real-time PCR, the cells were seeded at a density of 3 × 10^6^ cells/ml in 100-mm tissue culture dishes. In all cases, the cells were maintained in DMEM-S medium and cultured overnight at 37°C in a humidified atmosphere of 95% air and 5% CO_2_. Afterward, the medium was replaced with fresh culture medium (DMEM-S). Then the cells were either not treated (Untreated Control Group [UCG]) or treated with PTX, CIS, or PTX in combination with CIS (PTX + CIS). In the case of the PTX + CIS group, the cells were first exposed to PTX for 1 h; after this time CIS was added to the cell cultures.

### Dose-Response Curves for IC50 Determination

Dose-response curves were generated for CIS and PTX to determine the inhibitory concentration to achieve 50% cell death (IC50 value) for each cervical cancer cell line. Cytotoxicity was determined by TOX6-1KT SulfoRhodamine B (SRB) Cell Cytotoxicity Commercial Kit (Sigma-Aldrich) following the manufacturer’s instructions. Briefly, the cells were incubated in the absence or presence of different concentrations of CIS (1, 10, 50, 100, and 200 µM) or PTX (2, 4, 6, 8, 10, and 12 mM) for 24, 48, 72, and 96 h; after this, we evaluated the cytotoxicity. Then, absorbance was measured at 510 nm in a microplate reader (Synergy HT Multi-Mode Microplate Reader; Biotek, Winooski, VT, USA). All experiments were carried out in triplicate. IC50 values were calculated based on concentration–effect of the relationships generated by Prism GraphPad software (ver. 8). The Resistance ratio (Rr) was calculated utilizing the following formula: Rr = IC50 of drug-resistant cells/IC50 of parental cells. The IC50 values were derived from the dose-response profile.

### Cell Viability

Parental and resistant cervical cancer cell lines were seeded on 96-well plates (2 × 10^4^ cells/well) and treated with PTX (4 mM), CIS (30 µM), or PTX + CIS (4 mM + 30 µM) for 24 h. According to the manufacturer’s instructions, cell viability was assessed using the WST-1 assay (Commercial Kit; Sigma-Aldrich). The WST-1 reagent was added 3 h before the end of the incubation period. Absorbance was measured at 450 nm in a microtiter plate reader. All of the readings were normalized to the UCG, and the UCG was considered as 100% live cells.

### Assessment of Apoptotic DNA Fragmentation in SiHaP and SiHaCIS-R Cells

Apoptotic DNA fragmentation is a crucial feature of apoptosis. Therefore, internucleosomal DNA fragmentation was quantitatively assayed by the antibody-mediated capture and detection of cytoplasmic mononucleosome- and oligonucleosome-associated histone‒DNA complexes (Cell Death Detection ELISA^PLUS^ Kit; Sigma-Aldrich). Briefly, SiHaP and SiHaCIS-R cells were cultured in 96-well plates and treated with CIS (30 µM), PTX (4 mM), or PTX + CIS (4 mM + 30 µM) for 24 h. Afterward, the cell culture supernatants were removed. The cells were resuspended in 200 µl of the lysis buffer™ and lysed directly in the wells. Cell lysates were then centrifuged (1,200 rpm, 10 min), and the cytoplasmic fraction (20 µl) was employed to determine DNA fragmentation according to the manufacturer’s standard protocol. Subsequently, absorbance was measured at 405 nm (490-nm filter as a reference wavelength) in a microplate reader. In the DNA fragmentation test, the rate of apoptosis is reflected by the enrichment (fold increase) of mono- and oligonucleosomes accumulated in the cytoplasm. DNA fragmentation was calculated according to the following formula: Rate of Apoptosis = Absorbance of Sample Cells/Absorbance of UCG.

### Determination of Caspase-3, Caspase-8, and Caspase-9 Activity in SiHaP and SiHaCIS-R Cells

To evaluate caspase-3, -8, and -9 activity in SiHaP and SiHaCIS-R cells after the cells were treated with PTX (4 mM), CIS (30 µM), or PTX + CIS (4 mM + 30 µM) for 24 h, caspase-8 activity was determined by flow cytometry using the M30 CytoDEATH™ monoclonal antibody (Roche Mannheim, Germany). In brief, the cells were harvested and stained with the M30 antibody according to the manufacturer’s instructions. For each sample, at least 20,000 events were acquired in a FACSAria I Cell Sorter (BD Biosciences, San Jose, CA, USA), and the data were analyzed with FlowJo ver. 7.6.5 software (Tree Star, Inc., OR, USA). Caspase-3 and -9 activities were measured using the active Caspase-3 and Caspase-9 ELISA Kit (Abcam, Cambridge, UK). After 24 h of treatment, the cells were harvested and washed twice with PBS. According to the manufacturer’s instructions, the cells were resuspended in lysis buffer (Standard Cell Fractionation Buffer; Abcam, Cambridge, UK) containing a cocktail of protein inhibitors (Complete™, Mini, EDTA-Free; Roche-Diagnostics). Protein concentrations were determined using the Bradford assay (Bio-Rad). An equal amount of protein (100 µg) from each sample was analyzed according to the manufacturer’s instructions. Finally, absorbance was measured at 405 nm in a microplate reader. The results of caspase-3, -8, and -9 activity are expressed as fold changes in comparison with UCG.

### Determination of the Cleavage of PARP-1 in SiHaP and SiHaCIS-R Cells

SiHaP and SiHaCIS-R cells were treated with PTX (4 mM), CIS (30 µM), or PTX + CIS (4 mM + 30 µM) for 24 h. After the incubation period, the cells were harvested and washed with PBS twice. Then we added 300 µl of RIPA buffer (0.5% deoxycholate, 0.5% NP-40, 0.5% SDS, 50 mM Tris pH 8.0, and 150 mM NaCl) with the Complete Mini EDTA-free Protease Inhibitor Cocktail (Roche Applied Science, Penzberg, DE, USA), and cell suspensions were incubated on ice for 30 min. The lysates were sonicated (5 min, high level, 30 s on-off time interval) with the Bioruptor Sonicator (Diagenode, Liège, Belgium). Protein extracts were obtained after 30 min of incubation at 4°C and 12 min of centrifugation at 12,000 rpm/4°C. The Bradford Assay Kit (Bio-Rad) was used to determine the concentration of proteins in the samples. PARP-1 was evaluated in the cell proteins by Western blotting according to the following protocol. Samples containing 40 µg of total protein were resolved utilizing 14% SDS-PAGE. For immunoblot analyses, the proteins were transferred onto a PVDF membrane (0.2-µm pore) and then blocked with 1 × Western Blocking Reagent (Odyssey™) under agitation for 1 h at room temperature. The immunodetection of PARP-1, PARP-1 cleavage fragments, and β-actin was performed using anti-PARP-1 (1:1,000). The PARP-1 antibody detects endogenous levels of full-length 116 kDa PARP-1, as well as the large 89-kDa fragment (Cell Signaling Technology^®^, Danvers, MA, USA), and the anti-β-actin antibody (1:1,000, Abcam) overnight at 4°C under agitation and protected from light. The membranes were washed and probed with LI-COR IRDye 800 secondary antibodies for 1 h at room temperature. Then the washed membranes were scanned with an Odyssey™ Infrared Imagin System (LI-COR Biotechnology, Lincoln, NE, USA). The Optical Density (OD) of each Western blot lane was measured using Image Studio Lite ver. 5.2.5; a correction was made among the actin values according to the LI-COR standardization guide. The density of PARP-1 bands (total PARP-1 and PARP-1 cleavage fragments) was normalized with their corresponding β-actin value. To determine the fold change, the results of total PARP-1 or PARP-1 fragments were divided by the values of the UCG group.

### Assessment of Bcl-2, Bcl-XL, and the Phosphorylation of p65 in SiHaP and SiHaCIS-R Cells

To determine the expression of Bcl-2, Bcl-XL, and the phosphorylation of p65, SiHaP and SiHaCIS-R cells were treated with PTX (4 mM), CIS (30 µM), or PTX + CIS (4 mM + 30 µM) for 1 h (to measure phosphorylated p65) or for 24 h (to evaluate Bcl-XL and Bcl-2 expression). The staining procedures were performed according to the protocol for the detection of proteins or of activation of the phosphorylation state by flow cytometry. We used the following antibodies: Alexa Fluor-647 mouse anti-human Bcl-2; Alexa Fluor-647 mouse anti-human Bcl-XL proteins (Santa Cruz Biotechnology, Santa Cruz, CA, USA), and Alexa Fluor-647 mouse anti-human NF-κB p65 (BD Biosciences). Appropriate isotype controls were used for each parameter to adjust background fluorescence. The results are represented as the Mean Fluorescence Intensity (MFI) of Bcl-2, Bcl-XL proteins, and phosphorylated p65. For each sample, at least 20,000 events were acquired in a FACSAria I Cell Sorter (BD Biosciences), and data were analyzed with FlowJo ver. 7.6.5 software.

### Cadmium Chloride Cytotoxicity Assay

The cytotoxic activity of CdCl_2_ was determined using the SRB assay. In brief, SiHaP and SiHaCIS-R cells were seeded in 96 multi-well plates with DMEM-S medium and allowed to attach to the wells overnight. After 24 h, the medium was replaced with fresh medium (DMEM-S) and the cells were either not treated or treated with 4 mM PTX. These cells were then exposed to different concentrations of CdCl_2_ (0, 5, 20, 50, 70, 85, 100, 150, and 200 µM) and incubated for 96 h. Afterward, absorbance was measured at 510 nm using a microplate reader. Each experiment was performed in triplicate and repeated more than three times. IC50 values were calculated based on the concentration–effect relationships generated by Graph Pad Prism GraphPad software (ver. 8). The Resistance ratio (Rr) was calculated utilizing the following formula: Rr = IC50 of drug-resistant cells/IC50 of parental cells.

### Evaluation of GSH Levels in SiHaP and SiHaCIS-R Cells

Cellular GSH content in SiHaP and SiHaCIS-R cells was determined using the GSH Assay Kit (BioVision, Mountain View, CA, USA) according to the manufacturer’s instructions. Briefly, SiHaP and SiHaCIS-R cells were treated with PTX (4 mM), CIS (30 µM), or PTX + CIS (4 mM + 30 µM) for 24 h. After each treatment, the cells were harvested with PBS containing 5 mM EDTA and washed with ice-cold PBS twice. Cellular GSH was then extracted using 1 ml of ice-cold glutathione buffer™ and incubated on ice for 10 min. Then cold 5% sulfosalicylic acid (1,000 µl) was added, followed by a 10-min incubation step at 4°C with occasional shaking. After homogenization, the solution was centrifuged at 1,200 rpm for 10 min at 4°C. Sulfosalicylic acid was removed, and total GSH in the cell extracts was measured (17). Protein concentration was determined using the Bradford assay (Bio-Rad Laboratories, Inc., CA, USA). Finally, absorbance was measured at 412 nm in a microplate reader (Synergy HT Multi-Mode Microplate Reader). GSH concentration was determined by comparison with a standard curve and expressed as ng of GSH/10^6^ cells.

### Platinum Accumulation in SiHaP and SiHaCIS-R Cells

The accumulation of Pt in SiHaP and SiHaCIS-R cells was assessed by Quantitative-Inductively Coupled Plasma-Mass Spectrometry (Q-ICP-MS, VGElemental PQ3, Institute of Geophysics, National Autonomous University of Mexico, UNAM). Briefly, SiHaP and SiHaCIS-R cells were incubated with CIS (100 µM), PTX (4 mM), or their combination PTX + CIS (4 mM + 100 µM) for 5, 30, or 60 min. Immediately after this time, the cell monolayers were washed three times with ice-cold PBS (pH 7.6). The cells were then scraped and washed with PBS prior to resuspension in 1 ml of PBS and then placed on ice. The samples were then digested in 70% HNO_3_ before measuring the concentration of Pt. Pt levels were normalized to the protein content. Determination of the protein content was assessed using the Bradford method (Dc Protein Kit; BioRad Laboratories, Inc., Hercules, CA, USA). The cellular concentration of Pt is expressed as Pt ng/mg total protein.

### Determination of Gene Expression by qPCR in SiHaP and SiHaCIS-R Cells

For the qPCR analysis of gene expression (GSH, and efflux and influx drug genes), SiHaP and SiHaCIS-R cells were treated with PTX (4 mM), CIS (30 µM), or PTX + CIS (4 mM + 30 µM) for 4 h. Afterward, total RNA was extracted using the GeneJET™ RNA Purification Kit (Thermo Scientific, Waltham, MA, USA) following the manufacturer’s instructions. Complementary DNA (cDNA) was synthesized from 5 μg of total RNA using the Transcriptor First Strand cDNA Synthesis Kit (Roche Applied Science, Mannheim, Germany). qPCR reactions were conducted in a System Light Cycler^®^ 2.0 apparatus (Roche Applied Science) employing the DNA Master Plus SYBR Green I Kit (Roche Applied Science), as recommended by the manufacturer, with the addition of specific primers (5 pg of each, forward and reverse). The PCR program consisted of an initial 10-min step at 95°C, and 40 cycles of 15 sec at 95°C, for 5 sec at 60°C, and 15-sec cycles at 72°C. Analysis of the PCR products was performed using LightCycler^®^ software ver. 4.1 (Roche Applied Science). Data are expressed as relative normalized fold-change values following the E^ΔΔCp^ algorithm. The Ribosomal Protein L32 (*RPL32*) gene was used as reference gene. All reactions were performed in triplicate to avoid changes introduced by the operator. Sequences of the oligonucleotides used to amplify human *ATP7A*, *ATP7B*, *CTR1*, *MRP-2*, *GSR*, *GSS*, *GPX*, *MGST1*, and *RPL32* are shown in **Table 1**. They were designed using Oligo software ver. 6.0 (OLIGO, Colorado Springs, CO, USA) and commercially synthetized (Integrated DNA Technologies, Inc., Coraline, IA, USA). Gene sequences were obtained from the GenBank Nucleotide Database of the National Center for Biotechnology Information (NCBI) (http://www.ncbi.nlm.nih.gov).

**Table 1:** Primer pair sequences.

Gene	Direction	Primer pair sequences	GenBank Accession No.
***ATP7A***	Forward	5´CTG AAA TCT ATG GCC TTA GAA G 3´	NM_000052.7
	Reverse	5´CAT TGC TAC CCG TTT CC 3´	
***ATP7B***	Forward	5´CTT GGG ATA CTG CAC GGA CTT C 3´	NM_000053.4
	Reverse	5´CCT CAG CCA CTC ACG GTT TC 3´	
***CTR1***	Forward	5´TTG GCT TTA AGA ATG TGG ACC T 3´	NM_001184221.1
	Reverse	5´GAC TTG TGA CTT ACG CAG CA 3´	
***MRP-2***	Forward	5´GCT GGT GGC AAC CTG AGC ATA G 3´	NM_001316390.1
	Reverse	5´TGC AGT GGG CGA ACT CGT TT 3´	
***GSR***	Forward	5´CGA TGT ATC ACG CAG TTA CCA A 3´	NM_001195102.2
	Reverse	5´GGG TGA ATG GCG ACT GTG 3´	
***GSS***	Forward	5´CTG CCC CTA GCC GGT TTG 3´	NM_000178.4
	Reverse	5´GCT CTG AAA TGC ACT GGA CCA C 3´	
***GPX***	Forward	5´GGC CCA GTC GGT GTA TGC 3´	NM_001329790.2
	Reverse	5´TCT CTT CGT TCT TGG CGT TCT C 3´	
***MGST1***	Forward	5´TAT TCA TGG CTT TTG CAT CCT A 3´	NM_0012600511.1
	Reverse	5´GGC TCT GCG TAC ACG TTC TA 3´	
***RPL32***	Forward	5’GCA TTG ACA ACA GGG TTC GTA G 3’	NM_000994.4
	Reverse	5’ATT TAA ACA GAA AAC GTG CAC A 3’	

### Statistical Analysis

All experimental procedures were performed in triplicate and were repeated at least three times. The values represent the mean ± Standard Deviation (SD) of the obtained values. Statistical analysis was performed using the non-parametric Mann-Whitney *U* test to compare two groups. Differences were considered significant when *p* values were ≤0.05. Significant variations in gene-expression levels were considered when values were ≥30%. Data were analyzed using Prism ver. 8 GraphPad statistical software.

## Results

### Cytotoxicity and IC50 Determination

The CIS and PTX half maximal Inhibitory Concentration (IC50) in parental and CIS-R cervical cancer cells and the Rr are summarized in **Tables 2A** and **2B**. The IC50 value for CIS was higher in SiHaCIS-R cells than in HeLaCIS-R cells. Also, resistance to CIS increased approximately 2.98- to 3.68-fold at 24 and 96 h, respectively, in SiHaCIS-R cells (**Table 2A**). In contrast, the IC50 for PTX (**Table 2B**) was similar in parental HeLa and SiHa cell lines (4.50 and 4.30 mM, respectively, at 24 h) as well as in their resistant cell lines (4.44 and 4.50 mM, respectively, at 24 h). Furthermore, it is noteworthy that CIS-resistant cell lines (HeLa and SiHa cells) did not show resistance to PTX; the cytotoxic effect of this drug was similar in both cell lines. Together, these results demonstrated that SiHaCIS-R cells were more resistant to CIS than HeLaCIS-R cells, while no resistance to PTX was observed in either cell line (**Supplementary Figures 1** and **2**).

**Table 2A:** IC50 values for CIS (µM) in cervical cancer cells determined by the SRB assay.

Cell Lines	Time 24 h	Rr	Time 48 h	Rr	Time 72 h	Rr	Time 96 h	Rr
**HeLaP^a^**	14.00 ± 2.56		17.88 ± 5.21		15.60 ± 4.07		7.66 ± 2.78	
**HeLaCIS-R^b^**	22.85 ± 1.98	1.63**^+^**	17.47 ± 3.70	0.97	15.23 ± 2.80	0.97	7.83 ± 4.11	1.02
**SiHaP^a^**	33.20 ± 2.97		26.51 ± 4.33		18.82 ± 3.07		10.50 ± 3.13	
**SiHaCIS-R^b^**	99.00 ± 2.87	2.98*	80.00 ± 6.42	3.02*	70.77 ± 4.80	3.76*	38.74 ± 3.75	3.68*

The IC_50_ for CIS (µM) was determined by the SRB assay after drug treatment for 24, 48, 72, and 96 h. Resistance ratios (Rr) were calculated as the IC_50_ values of resistant cell line/IC_50_ value of the parental cell line. Each value is the mean ± Standard Deviation (SD) of three independent experiments, each performed in triplicate. ^+^p < 0.05 between HeLaP and HeLaCIS-R; *p < 0.01 between SiHaP and SiHaCIS-R cells. P^a^, Parental cells; CIS-R^b^, CIS-Resistant cells.

**Table 2B:** IC50 values for PTX (mM) in cervical cancer cells determined by SRB assay.

Cell Lines	Time 24 h	Rr	Time 48 h	Rr	Time 72 h	Rr	Time 96 h	Rr
**HeLaP^a^**	4.50 ± 0.12		4.44 ± 0.18		2.40 ± 4.07		2.00 ± 2.78	
**HeLaCIS-R^b^**	4.44 ± 0.18	0.98	3.88 ± 0.05	0.87	2.25 ± 2.80	0.93	2.10 ± 4.11	1.05
**SiHaP^a^**	4.30 ± 0.07		4.05 ± 0.15		3.50 ± 3.07		2.75 ± 3.13	
**SiHaCIS-R^b^**	4.50 ± 0.01	1.04	4.25 ± 0.04	1.13	3.00 ± 4.80	0.85	2.00 ± 3.75	0.72

The IC_50_ for PTX (mM) was determined by the SRB assay after treatment with the drug for 24, 48, 72, and 96 h. Resistance ratios (Rr) were calculated as the IC_50_ values of the resistant cell line/IC_50_ value of the parental cell line. Each value is the mean ± Standard Deviation (SD) of three independent experiments, each performed in triplicate. P^a^, Parental cells; CIS-R^b^, CIS-Resistant cells.

### Pentoxifylline Alone or in Combination With Cisplatin Decreased Cell Viability in HeLaCIS-R and SiHaCIS-R Cervical Cancer Cells

**Figure 1:**
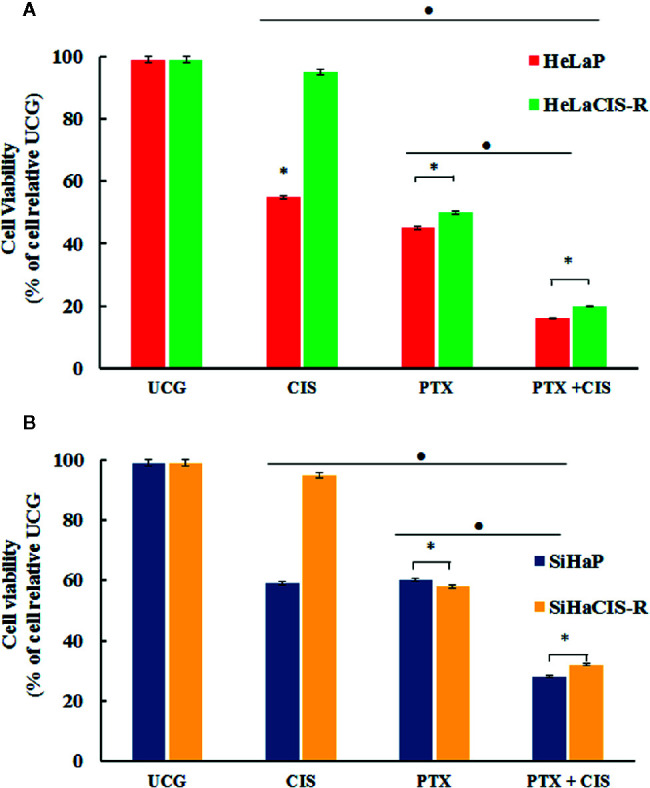
PTX decreases viability and induced sensitization to CIS treatment in resistant cervical cancer cell lines. Cell viability was assessed using the WST-1 assay **(A)**. HeLaP and HeLaCIS-R cell lines were treated with CIS (20 μM), PTX (4 mM), or PTX + CIS (4 mM + 20 μM) for 24 h **(B)**. SiHaP and SiHaCIS-R cells were treated with CIS (30 μM), PTX (4 mM), or PTX + CIS (4 mM + 30 μM) for 24 h. Data are expressed as the percentage of cells relative to the UCG. *p < 0.01 = statistical significance after comparison among the PTX, or CIS, or PTX + CIS groups in HeLaCIS-R cells or SiHaCIS-R *vs.* parental cells. ● *p* < 0.01 PTX + CIS *vs.* PTX group or CIS group in parental or resistant cells. UCG, Untreated Control Group; CIS, Cisplatin; PTX, PenToXifylline.

To assess whether PTX, CIS, or their combination (PTX + CIS) exert an effect on cell viability in parental and CIS-resistant cells, we performed the WST-1 assay. To this end, the cells were exposed to PTX or CIS alone, or in combination (PTX + CIS). We selected drug concentrations based on the IC50 values obtained for each cell line. According to our results, 4 mM PTX substantially decreased cell viability in HeLaCIS-R and SiHaCIS-R cell lines when compared to their respective UCG (**Figures 1A, B**; *p* < 0.01). Likewise, CIS reduced cell viability in HeLaP and SiHaP cells, but it had no effect on their CIS-resistant lines. However, when both cells lines were treated with the drugs in the combination (PTX + CIS), we observed a significant decrease in cell viability compared to their UCG or to the cells treated with either PTX or CIS alone (*p* < 0.01). Our results indicated that PTX possess a potent cytotoxic effect in HeLa cell lines compared to that which we observed in SiHa cells. Furthermore, parental and resistant SiHa cells revealed a higher and more stable CIS-resistant level in comparison with HeLa cells. Therefore, SiHa and SiHaCIS-R cells were used as a final model.

### PTX-Induced Sensitization of SiHaP and SiHaCIS-R Cells to Apoptosis and Caspase Activity

**Figure 2:**
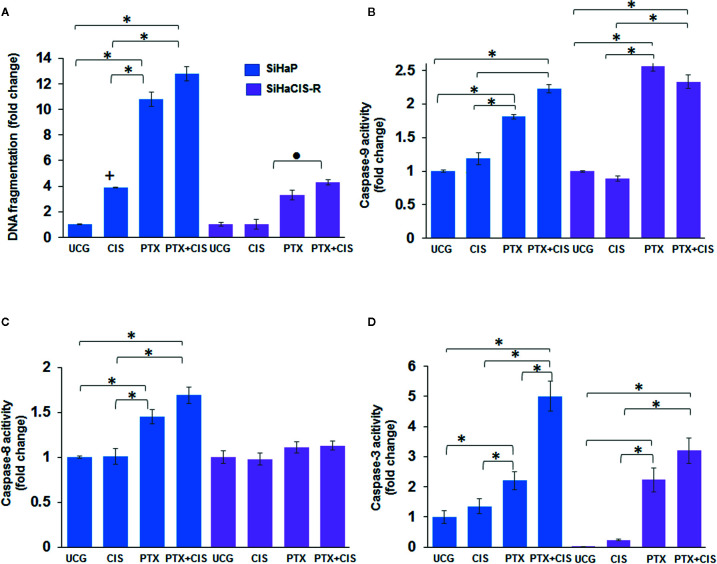
PTX-induced apoptosis and caspase activities in SiHaP and SiHaCIS-R cells. SiHaP and SiHaCIS-R cells were treated with CIS (30 μM), PTX (4 mM), or PTX + CIS (4 mM + 30 μM) for 24 h; then, apoptosis (DNA fragmentation **(A)**, caspase-9 **(B)**; caspase-8 **(C)**, and caspase-3 **(D)** activity were determined. Data are expressed as fold change relative to the UCG. *p < 0.001 = statistical significance after comparison among PTX, or PTX + CIS groups *vs.* CIS or UCG in SiHaP cell or SiHaCIS-R cells. **+***p* < 0.001 CIS group *vs.* UCG in SiHaP cells. ● *p* < 0.001 PTX + CIS group *vs.* PTX in SiHaCIS-R cells. UCG, Untreated Control Group; CIS, CISplatin; PTX, PenToXifylline.

We then wanted to evaluate whether pretreatment with PTX induced apoptosis in SiHaCIS-R cells. Thus, we measured DNA fragmentation and caspase-3, -8, and -9 activation in SiHaP and SiHaCIS-R cells treated with PTX, CIS, or PTX + CIS. According to the results shown in **Figure 2A**, when we exposed the cells to CIS, we observed that SiHaCIS-R cells were more resistant to DNA fragmentation in comparison to SiHaP cells. However, the treatment with PTX alone or in combination with CIS (PTX + CIS) increased DNA fragmentation in SiHaCIS-R cells in comparison with the treatment with CIS alone (*p* < 0.001). Regarding caspase activities (**Figure 2B**), we observed that CIS did not induce caspase-9 activation in SiHaCIS-R; while PTX alone or in combination with CIS induced higher activity of caspase-9 in SiHaP and SiHaCIS-R cells compared to the UCG or CIS treatment (*p* < 0.001). In contrast, PTX, alone or in combination, did not affect caspase-8 activity (**Figure 2C**) in SiHaCIS-R cells compared to the UCG. The increased activity of caspase-3 (**Figure 2D**) was also observed in both cells treated with PTX or PTX + CIS (*p* < 0.001) compared to the UCG or the CIS-treated group. Interestingly, when we only exposed the cells to CIS, we observed a considerably lesser activity of caspase-3 in SiHaCIS-R cells compared to parental cells also treated with CIS alone. Overall, these results suggested that PTX induced SiHaP and SiHaCIS-R cells sensitization to apoptosis, and that this pathway was mediated mainly by caspase-9 and -3 activation.

### PTX in Combination With CIS Strengthened PARP-1 Cleavage in SiHaCIS-R Cells

**Figure 3:**
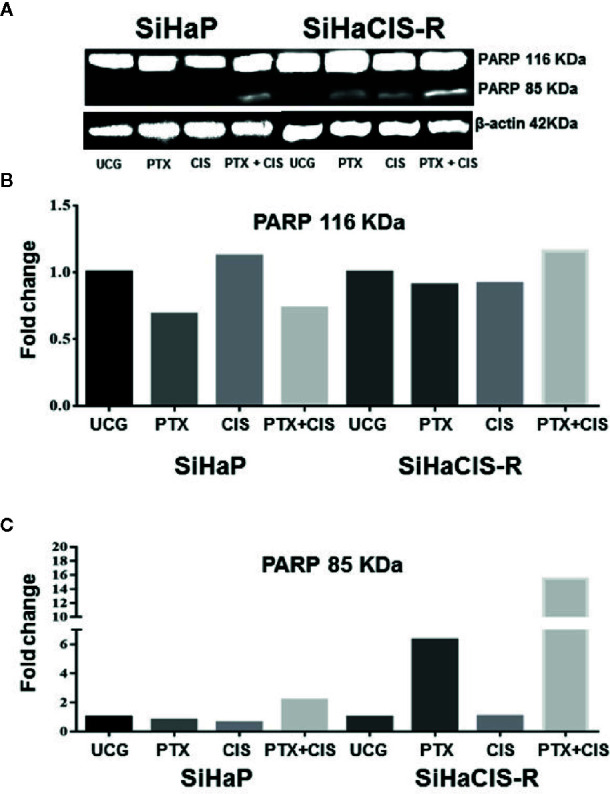
PTX in combination with CIS increased PARP-1 cleavage in chemoresistant SiHa cells. Western blot analysis of PARP-1 in SiHaP and SiHaCIS-R cells were treated with CIS (30 μM), PTX (4 mM), or PTX + CIS for 24 h. Then, PARP cleavage was evaluated by Western blot **(A)**. Densitometric analysis of total PARP-1 **(B)** and the cleavage of PARP **(C)** are shown. Relative density was calculated using Image Studio Lite ver. 5.2.5 (LI-COR Biotechnology) software. A representative example of three assays performed in triplicate is shown. β-Actin was used as a loading control. UCG, Untreated Control Group; CIS, CISplatin; PTX, PenToXifylline.

Caspase-3 plays a central role in the execution phase of apoptosis and is primarily responsible for the cleavage of PARP-1 during cell death. Caspase-3 activity and the cleavage of PARP-1 indicate the extent of apoptosis. Due to that the treatment with PTX or its combination with CIS increased caspase-3 activation, we evaluated the cleavage of PARP-1 in SiHaP and SiHaCIS-R cells treated with PTX, CIS, or PTX + CIS. According to the results depicted in **Figures 3A–C**, only the combination of PTX + CIS induced PARP-1 cleavage in SiHaP cells, while in SiHaCIS-R, PARP-1 cleavage was induced by the treatment with PTX or CIS alone and the combination of both (PTX + CIS). However, the most significant effect was observed when SiHaCIS-R cells were treated with PTX + CIS. These results indicate that PARP-1 cleavage comprises a critical pathway in the induction of apoptosis in resistant SiHa cells.

### PTX Decreased the Phosphorylation of P65 (NF-κB Subunit), Bcl-2, and Bcl-XL Anti-apoptotic Proteins Induced by Cisplatin Treatment in SiHaCIS-R Cells

**Figure 4:**
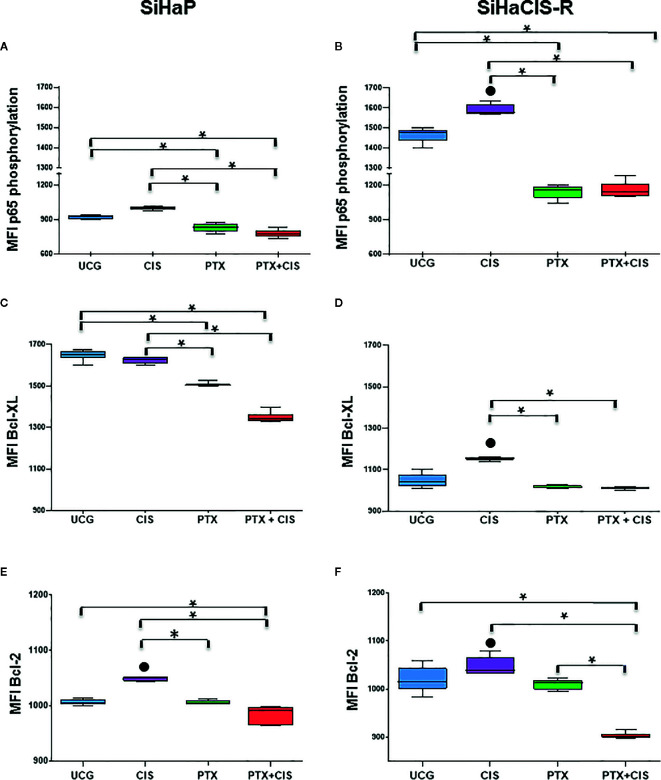
PTX decreased NF-κB/p65 phosphorylation and Bcl-2 and Bcl-XL anti-apoptotic proteins in SiHaP and SiHaCIS-R cells. SiHaP and SiHaCIS-R cells were treated with CIS (30 μM), PTX (4 mM), or PTX + CIS (4 mM + 30 μM) for 24 h to determine Bcl-XL and Bcl-2 expression. For the assessment of p65 phosphorylation, the cells were treated for 1 h. Data are expressed as the Mean Fluorescence Intensity (MFI) of p65 phosphorylation **(A, B)**, Bcl-XL **(C, D)**, and Bcl-2 **(E, F)** anti-apoptotic proteins. *p < 0.001 = statistical significance after the comparison between PTX group or the PTX + CIS group *vs.* CIS group or UCG groups in SiHaP cells or SiHaCIS-R cells. ● *p* < 0.001 CIS group *vs.* UCG group in SiHaP cells or SiHaCIS-R cells. UCG, Untreated Control group; CIS, CISplatin; PTX, PenToXifylline.

The anti-apoptotic functions of NF-κB, Bcl-2, and Bcl-XL play an important role in the development of resistance to cancer therapy. Therefore, we analyzed p65 phosphorylation (NF-κB subunit), Bcl-2, and Bcl-XL anti-apoptotic proteins in SiHaP and SiHaCIS-R cells treated with CIS, PTX, or PTX + CIS. As illustrated in **Figures 4A, B**, we observed that CIS induced an increase in p65 phosphorylation in SiHaP and SiHaCIS-R cells, this being more marked in SiHaCIS-R cells, whereas the opposite effect was observed in the cells treated with PTX or PTX + CIS (*p* < 0.001). PTX reduced constitutive activation of the p65 subunit in comparison with CIS or the UCG group (*p* < 0.001). A similar effect was observed in Bcl-2 and Bcl-XL anti-apoptotic proteins when SiHaP and SiHaCIS-R cells were treated with PTX or PTX + CIS (**Figures 4C–F**; *p* < 0.001). While CIS induced the expression of these anti-apoptotic proteins, PTX reduced it.

### SiHaCIS-R Is Cross-Resistant to Cadmium Chloride

Next, we evaluated whether SiHaCIS-R cells were cross-resistant to CdCl_2_ (**Table 3**). For this purpose, SiHaP and SiHaCIS-R cells were exposed to increasing concentrations of CdCl_2_ (5, 20, 50, 70, 85,100, 150, and 200 µM) for 96 h. SRB assays showed that SiHaCIS-R cells were cross-resistant to CdCl_2_. The IC50 values for CdCl_2_ in SiHaP and SiHaCIS-R cells were 65.53 and 191.00 µM, respectively. Thus, our results revealed that SiHaCIS-R cells were about 2.91-fold more resistant to CdCl_2_ compared to the SiHaP cell line, suggesting that these resistant cells may possess higher levels of metallothionein-like proteins. Likewise, the cells were treated with CdCl_2_, either alone or in combination with 4 mM PTX (PTX was added 1 h prior to CdCl_2_ exposure). Our data showed that the IC50 values were significantly lower in the PTX group than in the exclusively CdCl_2_-treated group. The Rr decreased from 65.53 to 1.75 µM in SiHaP cells and from 191.0 to 2.30 µM in SiHaCIS-R cells (*p* < 0.001). The Rr between both treated cells after CdCl_2_ exposure was only 1.31-fold. These results revealed that SiHaP and SiHaCIS-R cells responded similarly to PTX pretreatment, significantly reducing CdCl_2_ resistance (*p* < 0.001). Taken together, these data strongly indicate that pretreatment with PTX resensitized SiHaCIS-R cells to the effects of CIS and CdCl_2_, and they also suggest that PTX may be potentially used as a novel treatment strategy to overcome CIS resistance in cervical cancer.

**Table 3:** Resistance to Cadmium Chloride in SiHaP and SiHaCIS-R cervical cancer cell lines.

Cell Lines	CdCl_2_ (IC_50 µ_M)	Rr	CdCl_2_ (IC_50 µ_M) + PTX 4 mM	Rr
**SiHaP^a^**	65.53 ± 2.33		1.75 ± 0.02*	
**SiHaCIS-R^b^**	191.00 ± 0.28**	2.91	2.30 ± 0.18*	1.31

Resistance ratios (Rr) were determined as the IC_50_ values of the resistant cell line/IC_50_ value of the parental cell line. Each value is the mean ± Standard Deviation (SD) of three independent experiments, each performed in triplicate. *p < 0.001 SiHaP and SiHaCIS-R cells treated with PTX vs. SiHaP cells or SiHaCIS-R cells not treated with PTX; **p < 0.05 SiHaCIS-R cells vs. SiHa^P^ cells. P^a^, Parental cells; CIS-R^b^, CIS-Resistant cells.

### PTX Decreased the Glutathione Levels Induced by CIS in Parental and Chemoresistant SiHa Cells

**Figure 5:**
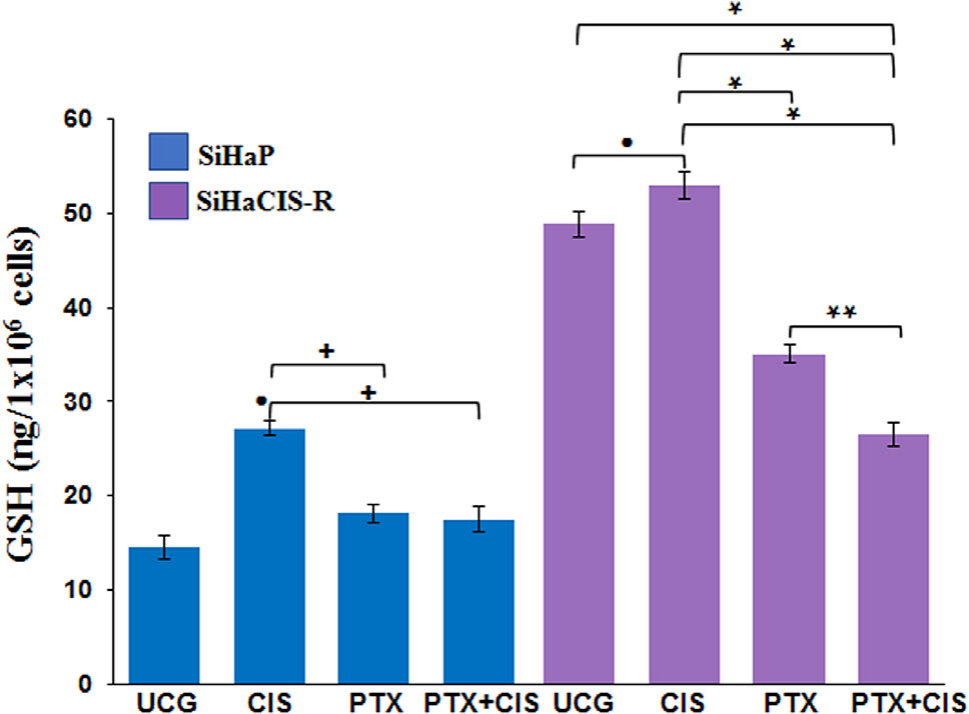
PTX decreased glutathione levels induced by CIS in parental and chemoresistant SiHa cells. SiHaP and SiHaCIS-R cells were treated with CIS (30 μM), PTX (4 mM), or CIS + PTX for 24 h; then, the glutathione levels were determined. Data are expressed as ng of GSH/10^6^ cells. *p < 0.001 = statistical significance after the comparison between PTX group or PTX + CIS group *vs.* CIS group or UCG in SiHaCIS-R cells. **+** *p* < 0.001 PTX group or PTX + CIS group *vs.* CIS group in SiHaP cells. **p < 0.001 PTX + CIS group *vs.* PTX group in SiHaCIS-R cells. ● *p* < 0.001 CIS group *vs.* UCG group in SiHaCIS-R or SiHaP cells. UCG, Untreated Control Group; CIS, CISplatin; PTX, PenToXifylline.

To study the mechanisms implicated in the sensitization of CIS-resistant SiHa cells to CIS treatment after PTX exposure, we decided to evaluate the intracellular levels of total GSH in parental SiHa cells and in CIS-resistant SiHa cells. As presented in **Figure 5**, baseline GSH levels were higher in SiHaCIS-R cells than in SIHaP cells. However, when both cells were exposed to 30 μM CIS for 24 h, we observed an increase in GSH levels compared with the UCG (*p* < 0.001). Contrariwise, it is important to note that when the cells were treated with PTX alone or in combination with CIS (PTX + CIS), we observed a significant reduction in GSH levels (*p* < 0.001). These results demonstrated that high GSH activity provides an additional basis for resistance to CIS and that PTX reduced GSH levels, thus sensitizing SiHaCIS-R cells to cell death.

### PTX Increased the Accumulation of Pt in SiHaP and SiHaCIS-R Cells

Pt drugs are the most powerful and commonly used cancer chemotherapeutics. However, the reduction of Pt accumulation is an important factor associated with the reduced efficiency of Pt-based drugs. Therefore, we wanted to know whether PTX could modify Pt accumulation after exposure to 100 µM CIS. **Table 4** presents the time-course and accumulation of Pt in SiHaP and SiHaCIS-R cells. In SiHaP cells, Pt accumulated time dependently until 1 h after drug exposure.

**Table 4:** Accumulation of Platinum (Pt) in SiHaP and SiHaCIS-R cervical cancer cell lines exposed to Cisplatin and pretreated or not with PenToXifylline.

Cell Line	Cisplatin exposure time	Pt concentration ng/mg total protein mean ± SD		Fold	p
		*PTX (−)*	*PTX 4 mM (+)*		
**SiHaP^a^**					
	5´	986.19 ± 8.3	586.40 ± 5.1	0.60	<0.001
	30´	440.00 ± 2.9	936.20 ± 7.1	2.13	<0.001
	60´	1016.00 ± 6.2	1457.00 ± 15.2	1.43	<0.001
**SiHaCIS-R^b^**					
	5´	573.47 ± 4.1	506.00 ± 3.7	0.88	<0.001
	30´	492.00 ± 3.2	908.00 ± 5.6	1.84	<0.001
	60´	670.00 ± 0.03	1340.00 ± 11.1	2.00	<0.001

SiHaP and SiHaCIS-R cells were incubated during 1 h with CIS (equimolar 100 µM), and total intracellular levels of Pt were determined by ICP-MS. The results represent the mean ± Standard Deviation (SD) of three independent experiments, each performed in triplicate. p < 0.001 SiHaP or SiHaCIS-R cells pretreated with 4 mM PTX vs. SiHaP or SiHaCIS-R cells not pretreated with PTX. The Mann-Whitney U test was used to compare differences between the groups. P^a^, Parental; CIS-R^b^, CIS-Resistant cells.

In comparison with parental cells, the accumulation of Pt in SiHaCIS-R cells did not achieve significant increase after 1 h of exposure to the drug (p < 0.001). Our results showed a significant reduction in the cellular retention of Pt in SiHaCIS-R cells compared to SiHaP cells. Moreover, it is important to stress that when SiHaP and SiHaCIS-R cells were preincubated with 4 mM PTX (prior to CIS treatment), we observed that the accumulation of intracellular Pt increased similarly in both cell lines. When we compared Pt accumulation in the cells treated with CIS alone *vs.* the cells treated with the combination of PTX + CIS, we also observed significant differences in the accumulation of Pt (*p* < 0.001) (**Table 4**). These findings suggest that PTX increased cellular Pt accumulation in SiHaCIS-R as compared to the parental cells.

### PTX Downregulated Efflux Pump, Multidrug Resistance, and Glutathione Genes in SiHaP and SiHaCIS-R Cells

**Figure 6:**
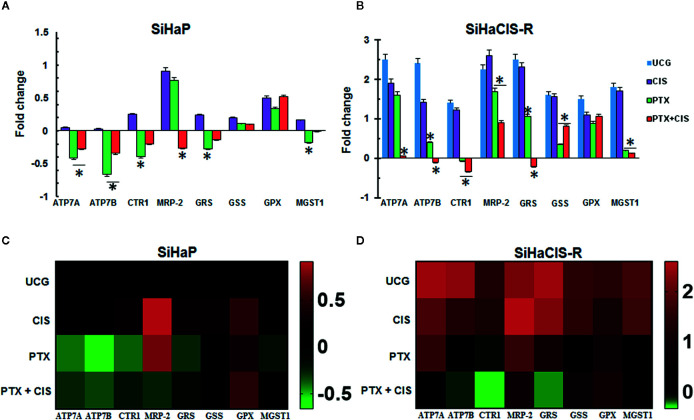
PTX downregulated efflux pump, multidrug resistance, and glutathione genes in SiHaP and SiHaCIS-R cells **(A, B)**. SiHaP and SiHaCIS-R cells were treated with CIS (30 μM), PTX (4 mM), or PTX + CIS (4 mM + 30 μM) for 4 h. Efflux *(ATP7A*, *ATP7B*, and *MRP-2)*, influx (*CTR1)*, *and Glutathione* genes (*GRS*, *GSS*, *GPX*, *and MGST1)* were evaluated by qPCR. *RPL32* was used as a reference *gene.* *p < 0.05 = statistical significance, comparisons were performed between PTX or PTX + CIS *vs.* CIS or UCG groups. Data are expressed as fold changes relative to the UCG of SiHaP cells **(C, D)**. Heat Maps of efflux *(ATP7A*, *ATP7B*, and *MRP-2*), influx (*CTR1*), *and glutathione* genes *(GRS*, *GSS*, *GPX*, and *MGST1*) expressed in SiHaP and SiHaCIS-R cells treated with PTX, CIS, or PTX + CIS.

To determine the possible involvement of the mechanisms associated with CIS resistance in SiHaCIS-R cells, we evaluated the expression pattern of efflux (*ATP7A*, *ATP7B*, and *MRP-2*), influx (*CTR1*), and GSH (*GSR*, *GSS*, *GPX*, and *MGST1*) genes by qPCR. All of these genes were readily detectable in both cells, with a differential expression between SiHaCIS-R and SiHaP cells (**Supplementary Table 1**). The results revealed that, under baseline conditions, the *ATP7A*, *ATP7B*, *CTR1*, *MRP-2*, *GSR*, *GSS*, *GPX*, and *MGST1* genes are significantly overexpressed (*p* < 0.05) in SiHaCIS-R cells (values comprise those between 1.4- and 2.5-fold), compared to parental cells. These data indicate that Pt transporters and GHS are probably involved in the resistant phenotype of SiHaCIS-R cells and in the remaining drug efflux pumps such as *MRP-2*. However, when the cells were treated with PTX, this drug induced the downregulation of the *ATP7A*, *ATP7B*, *CTR1*, *MRP-2*, and *GSR* genes in both cervical cancer cell lines (**Figures 6A, B**). It is important to note that, when we used PTX + CIS in SiHaCIS-R cells, we observed a stronger downregulation of the *ATP7A*, *ATP7B*, *CTR1*, *MRP-2*, *GSR*, and *MGST1* genes (*p* < 0.05). These results indicate that these genes were differentially expressed in parental and chemoresistant SiHa cells, and that PTX treatment induced the downregulation of the genes related to CIS chemoresistance (**Figures 6C, D**).

## Discussion

CIS remains one of the most utilized and effective anticancer drugs for the treatment of recurrent and advanced cervical cancer. However, over time, resistance can be developed even when the tumors are initially susceptible to this drug (3). Several mechanisms are involved in the development of Cisplatin resistance, such as decreased cellular drug accumulation, enhanced drug inactivation, and augmented DNA repair (18). These mechanisms are intrinsic or acquired after treatment cycles and confer the Cisplatin-resistant phenotype of cancer cells (19). Therefore, to overcome CIS resistance in cervical cancer, it is important to develop novel therapeutic modalities. Among the different options, the administration of CIS, in combination with other drugs that help to reverse the CIS-resistant phenotype, may be a promising alternative for patients who exhibit resistance.

In the present study, we selected two cervical cancer cell lines, HeLa and SiHa, as our initial model to generate CIS resistance. They are derived from human cervical carcinomas, and although both cell lines resulted from HPV-mediated transformation (HeLa is HVP-18 positive, whereas SiHa is HPV-16 positive), the levels of CIS resistance observed after exposure to the drug were different. We found that the SiHaCIS-R cell line was more resistant to the effects of CIS than HeLaCIS-R cells. Likewise, several studies have shown that chemoresistance is intimately linked to cell type and to the inherent self-response capacity of the cells in terms of prolonged exposure to the drug. These findings are consistent with those of a previous study where was reported that HeLa cells are more sensitive to Cisplatin than are CaSki cells and SiHa cells (20).

Therefore, we decided to use only the SiHaCIS-R cell line as a final model to evaluate the effects of PTX. However, despite the agreement in our results herein, there may be some possible limitations in this study. HeLaCIS-R cells did not maintain a completely stable resistance despite having been subjected to the same protocol as SiHa cells; thus, we selected SiHa cell lines to work. Interestingly, HeLaCIS-R cells were sensitive to the effects of PTX alone or in combination with CIS in a similar manner to that of SiHaCIS-R cells. Together, these observations suggest that PTX could improve the sensitivity of CIS in different types of cervical cancer cell lines that are less sensitive to CIS.

In this study, we showed that SiHa cells with an acquired CIS-resistant phenotype were refractory to CIS effects *in vitro*. Overall, our data revealed that the acquisition of CIS resistance is correlated with Pt accumulation, elevated GSH expression, phosphorylation of NF-κB/p65, and the inhibition of apoptosis. In this respect, our results are consistent with those of other authors who have also demonstrated that resistance to CIS in SiHa cells is caused by the overactivation of the pathways that regulate the intracellular drug uptake and the inhibition of apoptosis. It was reported that the simultaneous administration of two or more chemotherapeutic drugs with different mechanisms of action minimized the development of CIS resistance by targeting different signal transduction cascades or enabling the incorporation of CIS (21). In previous studies, we evaluated the effect of PTX treatment on cervical cancer cells and observed that PTX sensitizes HeLa and SiHa cells to the antitumor effects of CIS by the inhibition of NF-κB (22). However, whether PTX can resensitize CIS-resistant cells remained to be determined. In the present study, we found that PTX, *per se*, reversed CIS resistance in SiHaCIS-R and HeLaCIS-R cervical cancer cells. This reversal of CIS resistance was more evident when the cells were pretreated with PTX prior to administering CIS; the effect was even higher than that observed when both molecules were administered.

The primary mechanism by which CIS exerts its action through the formation of adducts with DNA. However, it was reported that, once inside the cells, CIS interacts with other molecules in addition to DNA, including sulfur-containing macromolecules such as MetalloThioneins (MT) and GSH, which sequester CIS and remove it from the cells, leading to the development of CIS resistance. Thus, to elucidate the role of MT in the acquisition of CIS resistance, we analyzed sensitivity to CdCl_2_ in the cell lines, due to that cells containing excessive amounts of MT are more resistant to CdCl_2_ toxicity (23, 24). In our study, we observed that SiHaCIS-R cells were more resistant to CdCl_2_ (about 2.91-fold) compared to the SiHaP cell line. It is well known that MT binds to CdCl_2_. Thus, the overexpression of MT results in tolerance to Cadmium toxicity. It was also reported that GSH can participate in resistance to heavy-metal toxicity (25), due to that GSH depletion has been associated with increased Pt accumulation (26). When we evaluated the accumulation of Pt in SiHaP and SiHaCIS-R cells, we observed that SiHaCIS-R cells exhibit a decrease in Pt accumulation; this can explain their reduced susceptibility to Pt uptake. According to our results, baseline GSH levels were higher in SiHaCIS-R cells than in SiHaP cells.

Interestingly, when we treated the cells with PTX, we observed that GSH levels were reduced in resistant and parental SiHa cells. However, this effect was more evident in the groups of cells treated with both drugs (PTX + CIS). In this regard, it was reported that PTX induces the depletion of intracellular GSH (27). It is also known that the biosynthesis of GSH is ATP-dependent. The inhibition of phosphodiesterase activity by PTX decreases the levels of 5´AMP, limiting the formation of ATP. This may explain the reduction in GSH levels observed in SiHaP and SiHaCIS-R treated with PTX alone or in combination with CIS.

Furthermore, these results suggest that PTX can regulate the balance of GSH. It is important to note that we demonstrated that the treatment with PTX alone or in combination with CIS induced a significant increase in Pt accumulation (2-fold), as well as a significantly lower level of resistance to CdCl_2_. These data showed that MT and GSH could act as a line of defense that protects resistant cells against CIS cytotoxicity, playing an important role in drug efflux.

The NF-κB signaling pathway has been also implicated in chemoresistance (28) and in the positive regulation of the anti-apoptotic proteins Bcl-2 and Bcl-XL (29). Many reports have demonstrated the importance of these anti-apoptotic proteins in the development of resistance to CIS (30–32). However, we observed that Bcl-XL and Bcl-2 proteins were not related with resistance to CIS in cervical cancer cells, since the expression of Bcl-XL was even higher in parental SiHa cells than in resistant SiHa cells, whereas Bcl-2 expression was similar in both cell lines. However, it is noteworthy that PTX alone or in combination with CIS induced the reduction of both proteins in SiHaCIS-R cells and SiHaP cells. Interestingly, under baseline conditions, we observed an increase in p65 phosphorylation in SiHaCIS-R cells compared with parental SiHa cells. In this respect, it was shown that NF-κB is overactivated in cancer, and can regulate the pathways that mediate tumor-cell proliferation, survival, and angiogenesis (33). We also found that PTX treatment alone or in combination with CIS reduced the phosphorylation of p65 in both cell lines (parental or resistant SiHa cells). Additionally, we observed that the treatment with PTX + CIS increased PARP-1 cleavage in SiHaP and SiHaCIS-R cells, which potentiates the apoptotic effects of these drugs, due to that these mechanisms are involved in the induction of apoptosis in human cervical cancer cells (34). PTX exerts similar effects on other types of cancer, for example, on lymphocytes from patients with chronic lymphocytic leukemia, PTX induces DNA fragmentation and caspase-3 activation, and decreases NF-kB/p65 phosphorylation (35). Similarly, in triple-negative MDA-MB231 breast cancer cells, PTX induces apoptosis through NF-κB suppression (14). These data suggest that, in SiHaCIS-R cells, NF-κB/p65 survival pathways, together with elevated levels of GSH, could protect CIS-resistant cells from cell death. Our results are consistent with those of other studies that consider NF-κB inhibitors as valid drug targets in resistant cells (33, 36, 37). In general, our findings demonstrated that PTX sensitized SiHa cells that were chemoresistant to CIS treatment by destabilizing the mitochondrial pathway, which induced caspase-9 activation. We also found an important induction of caspase-3 activity with an increase in DNA fragmentation when resistant and sensitive cells were treated with PTX or with PTX + CIS. Likewise, NF-κB can promote the modulation of several genes related to chemoresistance (*MRP-2*), the efflux of drugs (*ATP7A* and *ATP7B* genes) (38, 39), or GSH systems (*GSR*, *GPX*, *GSS*, and *MGST1* genes) (40, 41). Our data revealed that messenger RNA (mRNA) expression levels of genes involving copper homeostasis (*ATP7A*, *ATP7B*, and *CTR1*) were significantly higher in SiHaCIS-R cells than those in parental cells.

This result suggests that *ATP7A* and *ATP7B* may contribute to CIS resistance in SiHaCIS-R cells. It was previously reported that the overexpression of *ATP7A* and *ATP7B* renders human cells resistant to copper and CIS (42). *ATP7A* and *ATP7B* are two copper-transporting P-type ATPases that participate in copper homeostasis and that have been implicated in Pt efflux (18, 43). Similarly, *GSR*, *GSS*, and *GPX* are involved in GSH systems. Their upregulation is associated with chemoresistance (44, 45). *CTR1* is related to a greater influx of CIS into the cells; our results indicated that there is a balance between the expression of *ATP7A/B* and *CTR1*. Furthermore, the downregulation of *ATP7A/B* genes observed in SiHaCIS-R cells treated with PTX may contribute to the sensitization of cells that are resistant to CIS, due to that PTX alone or in combination with CIS induced DNA fragmentation and caspase activity, as well as the decrease in the phosphorylation of p65 and in anti-apoptotic proteins (Blc-2 and Bcl-XL).

## Conclusions

PTX induced the sensitization of CIS-resistant SiHa cells to apoptosis by activating caspase-9 and -3 activity, reducing NF-kB/p65 phosphorylation, and increasing PARP-1 cleavage. In addition, we observed that the PTX effect decreased GSH levels and downregulated the expression of *ATP7A/B* and *GSR* genes. These results are consistent with our previous observations and confirm the concept of chemotherapy with rational basis. Therefore, PTX may be a potential candidate for use in cervical cancer therapy in combination with CIS, particularly in the treatment of patients who exhibit resistance.

## Data Availability Statement

The original contributions presented in the study are included in the article/**Supplementary Material**. Further inquiries can be directed to the corresponding author.

## Author Contributions

GH-F, PO-L ES-D, FS-I, and AM-C carried out experimental work. GH-F, AA-L, LJ-S, and PO-L performed the molecular study. AB-C, PO-L, and GH-F performed the statistical analysis, conceived and drafted the manuscript. All authors contributed to the article and approved the submitted version.

## Funding

This work was supported by a research grant (FIS/IMSS/PROT/G15/1415) from the Instituto Mexicano del Seguro Social (IMSS).

## Conflict of Interest

The authors declare that the research was conducted in the absence of any commercial or financial relationships that could be construed as a potential conflict of interest.

## Supplementary Material

Supplementary Figure 1 IC50 values for Cisplatin in cervical cancer cells were determined by the SRB assay. Comparison of the dose-response curves for CIS in HeLaCIS-R cells **(A)**, HeLaP cells **(B)**, SiHaCIS-R cells **(C)**, and SiHaP cells **(D)**. Each point represents the mean ± Standard Deviation (SD) of three independent experiments, each performed in triplicate.

Supplementary Figure 2 IC50 values for Pentoxifylline in cervical cancer cells were determined by the SRB assay. Comparison of the dose-response curves for PTX in HeLaCIS-R cells **(A)**, HeLaP cells **(B)**, SiHaCIS-R cells **(C)**, and SiHaP cells **(D)**. Each point represents the mean ± Standard Deviation (SD) of three independent experiments, each performed in triplicate.

Supplementary Figure 3 A representative example of a strategic analysis of p65 phosphorylation, Bcl-XL, and Bcl-2 expression by flow cytometry is depicted.
