# Supplementary figures and images for: Pentoxifylline Sensitizes Cisplatin-Resistant Human Cervical Cancer Cells to Cisplatin Treatment: Involvement of Mitochondrial and NF-Kappa B Pathways
Source: Front Oncol. 2020 Dec 16;10:592706. doi: 10.3389/fonc.2020.592706 (PMC7931705; doi:10.3389/fonc.2020.592706)

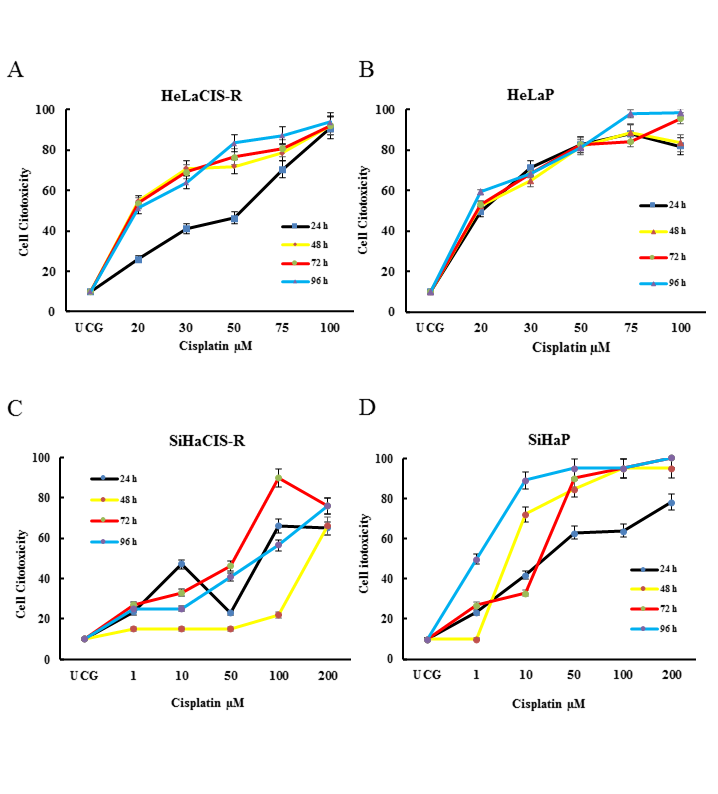

Supplement: Supplementary Figure 1 — IC50 values for Cisplatin in cervical cancer cells were determined by the SRB assay. Comparison of the dose-response curves for CIS in HeLaCIS-R cells (A), HeLaP cells (B), SiHaCIS-R cells (C), and SiHaP cells (D). Each point represents the mean ± Standard Deviation (SD) of three independent experiments, each performed in triplicate. [file Image_1.tif]

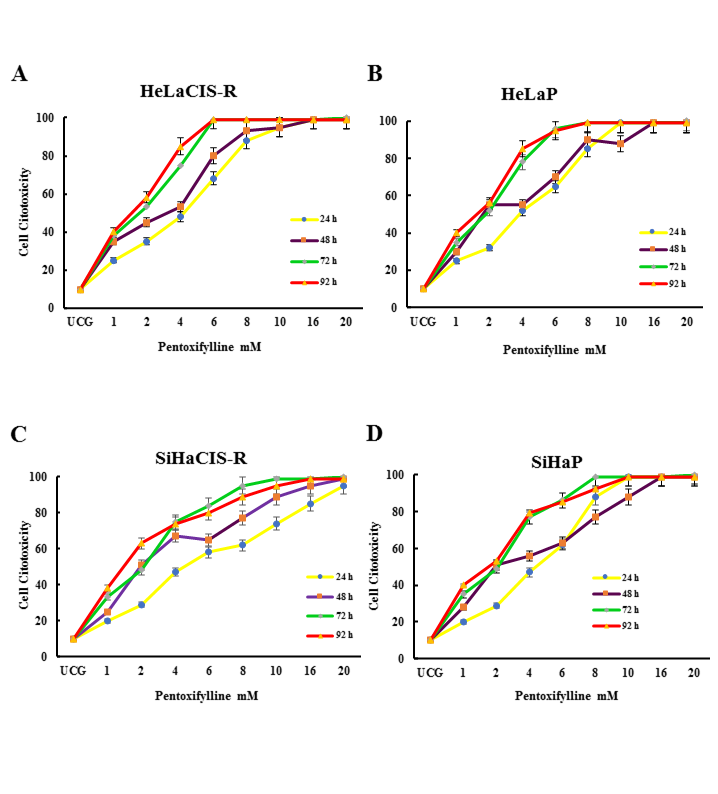

Supplement: Supplementary Figure 2 — IC50 values for Pentoxifylline in cervical cancer cells were determined by the SRB assay. Comparison of the dose-response curves for PTX in HeLaCIS-R cells (A), HeLaP cells (B), SiHaCIS-R cells (C), and SiHaP cells (D). Each point represents the mean ± Standard Deviation (SD) of three independent experiments, each performed in triplicate. [file Image_2.tif]

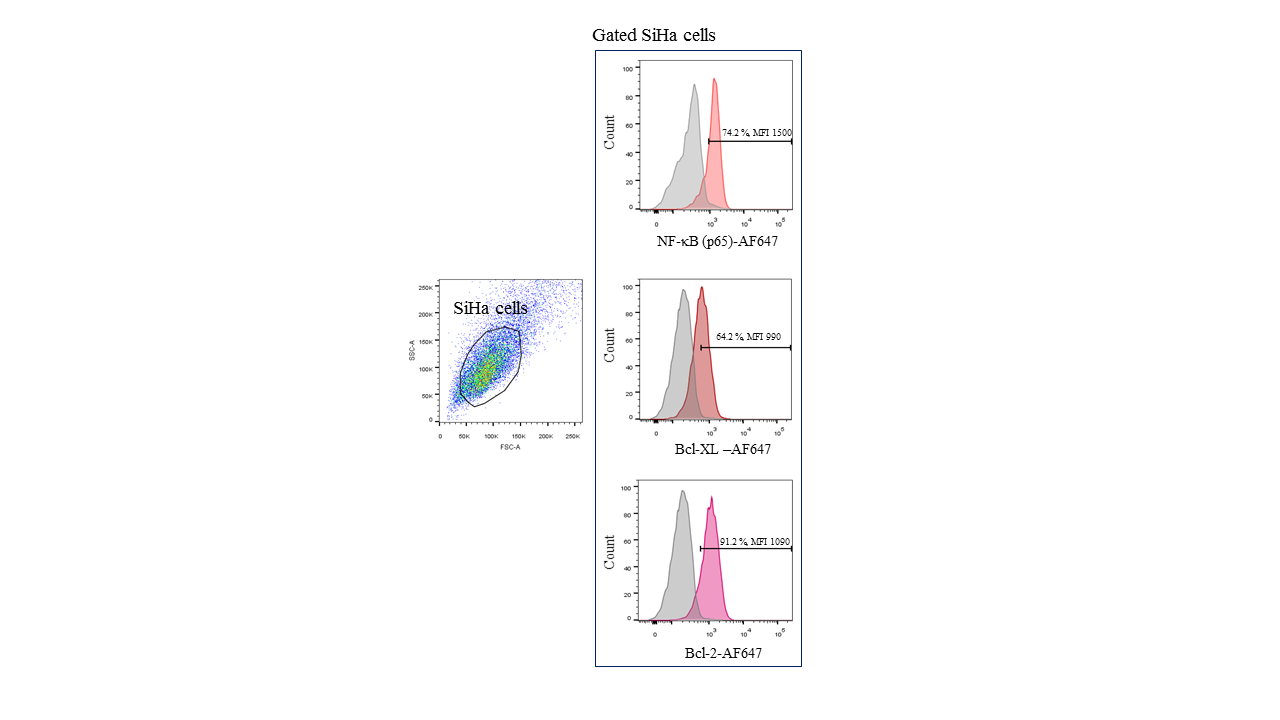

Supplement: Supplementary Figure 3 — A representative example of a strategic analysis of p65 phosphorylation, Bcl-XL, and Bcl-2 expression by flow cytometry is depicted. [file Image_3.tif]
