# Supplementary material for: Pentoxifylline Sensitizes Cisplatin-Resistant Human Cervical Cancer Cells to Cisplatin Treatment: Involvement of Mitochondrial and NF-Kappa B Pathways
Source: Front Oncol. 2020 Dec 16;10:592706. doi: 10.3389/fonc.2020.592706 (PMC7931705; doi:10.3389/fonc.2020.592706)
Supplement: Supplementary file 4 [file Table_1.docx]

**Supplementary Table 1.**

Relative levels of the mRNA expression of genes in SiHaCIS-R cells by real-time PCR

| **Gene** | **mRNA Fold-Change**  **in qPCR** |
| --- | --- |
| *ATP7A* | 2.50 |
| *ATP7B* | 2.40 |
| *CTR1* | 1.40 |
| *MRP-2* (*ABCC2*) | 2.25 |
| *GSR* | 2.45 |
| *GPX* | 1.50 |
| *GSS* | 1.60 |
| *MGST1* | 1.80 |

Comparison of relative mRNA expression levels in SiHaCIS-R in comparison with SiHaP cells
